# Experience in emergency management of first-episode immune thrombotic thrombocytopenic purpura over the past 21 years: a single-center retrospective study

**DOI:** 10.3389/fimmu.2025.1645558

**Published:** 2026-01-14

**Authors:** Liqian Zhang, Wenfeng Huang, Fengtao Yang, Lingjie Cao, Maojing Shi, Weibo Gao, Yuanyuan Pei, Jihong Zhu

**Affiliations:** Department of Emergency, Peking University People’s Hospital, Beijing, China

**Keywords:** clinical analysis, emergency management, immune thrombotic thrombocytopenic purpura, prognostic predictors, response to treatment

## Abstract

**Background:**

Immune thrombotic thrombocytopenic purpura (iTTP) is a rare but fatal hematologic disorder characterized by thrombocytopenia, microangiopathic hemolytic anemia, and multiorgan dysfunction. Early diagnosis and prompt treatment, including plasma exchange (PE), corticosteroids, and rituximab (RTX), are critical for improving outcomes. However, real-world emergency management experiences for first-episode iTTP remain understudied.

**Methods:**

This single-center retrospective study analyzed 96 patients with first-episode iTTP admitted to the emergency department of Peking University People’s Hospital between 2004 and 2024. Baseline characteristics, clinical features, treatment modalities, and outcomes were evaluated. Logistic and Cox regression analyses were conducted to identify predictors of clinical response and mortality.

**Results:**

Among the enrolled patients, the median age was 45 years and 54.2% were female. The comorbidities included rheumatologic and antoimmune diseases (28.1%) and cancer (11.5%). The mortality rate was 38.5% whereas the relapse rate was 13.6% in survival group. RTX administration increased over time (0% in 2004–2010 vs. 51.9% in 2018-2024) and was associated with improved survival (HR = 0.27, 95% CI: 0.11-0.66). Corticosteroid pulse therapy was an independent predictor of clinical response (OR = 2.8, 95% CI: 1.1-7.6). Independent predictors for mortality included age over 45 years (HR = 5.5, 95% CI: 2.1-14.3), pentad symptoms (HR = 3.5, 95% CI: 1.5-8.4), and lactate dehydrogenase over 1500 U/L (HR = 2.7, 95% CI: 1.2-5.8).

**Conclusions:**

This study highlighted the importance of early RTX and corticosteroid pulse therapy in the emergency management of first-episode iTTP. We also provide a risk stratification framework for emergency clinicians, guiding more prompt and effective management as well as personalized therapy.

## 1 Introduction

Thrombotic thrombocytopenic purpura (TTP) is a rare but life-threatening hematologic disease, with an incidence of 1 to 6 per million individuals (1, 2). Characterized by thrombocytopenia, microangiopathic hemolytic anemia (MAHA), and multiorgan dysfunction linked to ischemia caused by disseminated microvascular platelet-rich thrombi, TTP has a mortality rate of 10-31% (2, 3). The critical nature of TTP demands early recognition and prompt initiation of appropriate treatment, especially in the emergency setting.

The significant deficiency of ADAMTS13, a disintegrin and metalloproteinase with a thrombospondin type 1 motif, member 13, which is involved in preventing excessive platelet aggregation and microthrombus formation, leads to TTP (3–5). Hereditary or congenital TTP is caused by ADAMTS13 gene mutations and immune TTP (iTTP) results from anti-ADAMTS13 autoantibodies. Some clinical factors including autoimmune diseases, infections, and drugs are also related to reductions in ADAMTS13 and iTTP (2, 6). ADAMTS13 activity < 10% is the most accurate diagnostic criterion for iTTP and plasma exchange (PE), corticosteroids, rituximab (RTX), and caplacizumab are the first-line treatment options recommended by current clinical guidelines (1, 2, 7). Although ADAMTS13 is crucial for establishing a diagnosis of iTTP, it is not always available at every medical center, especially in emergency laboratories. In addition, there is always a contradiction between the insufficiency of the complex tests and the demand to initiate precise treatments promptly (8). More clinical experience is imperative to promote timely and effective diagnosis and treatment in real-world emergency cases involving iTTP.

Over the past 21 years, our center has been actively involved in the emergency management of iTTP patients. This retrospective study aims to summarize our experience, analyze the clinical characteristics, treatment modalities, and prognoses of first-episode iTTP patients presenting to our emergency department, with the hope of contributing to the existing body of knowledge and guiding future emergency treatment of iTTP.

## 2 Methods

### 2.1 Study design and population

**Figure 1:**
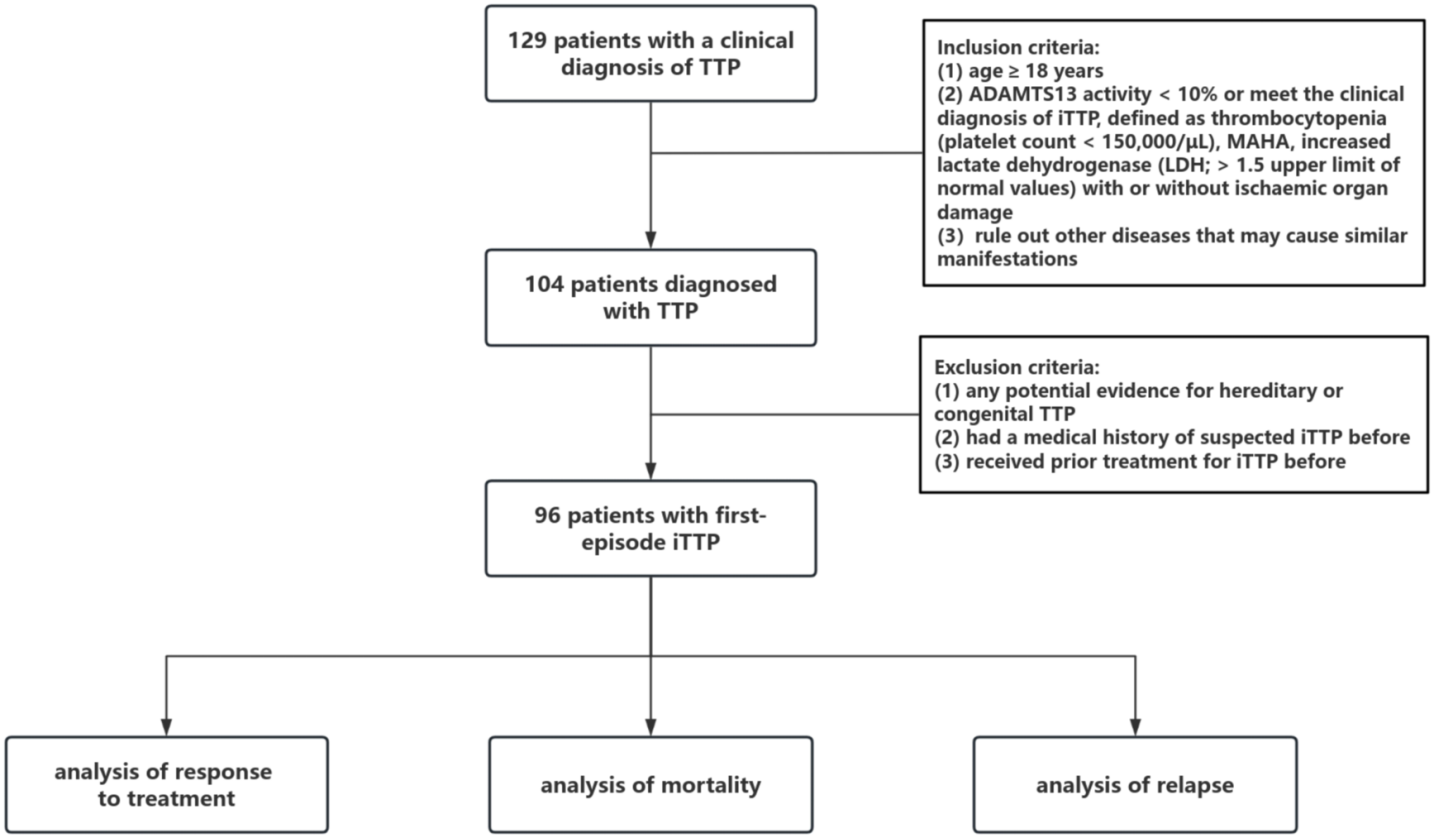
The study flow chart. TTP, thrombotic thrombocytopenic purpura; MAHA, microangiopathic hemolytic anemia; LDH, lactate dehydrogenase.

In this single-center retrospective study, we analyzed all iTTP patients referred to the department of emergency of Peking University People’s Hospital from January 2004 to December 2024. ICD-9-CM code 446.6 and ICD-10-CM code M31.1 were used to help identify a diagnosis of TTP in the electronic medical records system. The inclusion criteria were (1) age ≥ 18 years; (2) ADAMTS13 activity < 10% or meeting the clinical diagnosis of iTTP, defined as thrombocytopenia (platelet count < 150, 000/μL), MAHA, increased lactate dehydrogenase (LDH; > 1.5 upper limit of normal values) with or without ischemic organ damage and other diseases that may cause similar manifestations were ruled out (1, 9, 10). The exclusion criteria were as follows: (1) had any potential evidence of hereditary or congenital TTP (e.g., a definite family history of TTP or gene sequencing results of ADAMTS13); (2) had a previous medical history of suspected iTTP before; (3) had previously received prior treatment for iTTP before. The patient screening process is shown in **Figure 1**. The study protocol was approved by the Ethics Committee of Peking University People’s Hospital. Because data were collected retrospectively in this study, informed consent was exempt according to the institutional ethics regulations concerning the observational study nature of this study.

### 2.2 Data collection

The demographic and clinical characteristics were collected from the electronic medical records system at Peking University People’s Hospital and the data were anonymized and deidentified before analysis. The collected data included sex, age, etiological factors, clinical presentation, related examination results, intervention during the hospital stay and the response to treatment. Clinical response was defined by a sustained platelet count ≥ 150 × 10^9^/L and LDH < 1.5 times the upper limit of normal and no clinical evidence of new or progressive ischemic organ injury (11).

### 2.3 Follow-up and endpoints

Patients were followed for mortality and relapse through telephone interviews or outpatient visits. Relapse was defined as a decrease in platelets to < 150 × 10^9^/L with or without evidence of new ischemic organ injury after a clinical remission or ADAMTS13 levels < 20% after an ADAMTS13 complete or partial remission (7). Other causes of thrombocytopenia need to be ruled out.

### 2.4 Statistical analysis

Baseline features and hospital characteristics were summarized using descriptive statistics and compared between iTTP patients with favorable and unfavorable survival outcomes. Continuous variables are presented as the means ± standard deviations or medians (interquartile ranges). The Student’s t-test or the Mann-Whitney U nonparametric test was used to evaluate between-group differences. Categorical variables are expressed as frequencies and percentages, and group comparisons were tested via chi-square analysis or Fisher’s exact test. The effectiveness of therapeutic methods was assessed by Kaplan-Meier analysis and compared using the log-rank test. Univariate and multivariate logistic regression were employed to explore potential predictors of the response to iTTP treatment. Two sided *P* values < 0.05 were considered significant. Potential predictors of poor prognosis were analyzed using binary Cox regression and variables with *P* < 0.1 in the univariate analysis were included in a multivariate model. All the statistical analyses were performed with SPSS version 26 (IBM Inc., Armonk, NY, USA) and R version 4.2.2 (R Foundation, Vienna, Austria).

## 3 Results

### 3.1 Baseline characteristics and clinical features

During the study period, a total of 96 patients who visited our emergency center were ultimately included in this study, among whom 37 patients died during the follow-up duration. The median follow-up time in this study was 55.0 days. The maximum overall survival, which was defined as the earliest time from iTTP diagnosis to death, in people met mortality outcome was 43 days. The median age of the patients at diagnosis was 45.0 years, and 52 patients (54.2%) were female. Eleven patients (11.5%) had a history of cancer, 27 patients (28.1%) had comorbid rheumatologic and immunologic diseases, and 6 patients (6.3%) had a history of solid organ transplantation or hematopoietic stem cell transplantation. The baseline and clinical characteristics are summarized in **Table 1**.

**Table 1:** Baseline and clinical characteristics of patients in this study.

Variables	Overall (n = 96)	Survival (n = 59)	Death (n = 37)	*P*
Age, M (Q_1_, Q_3_) (years)	45.0 (32.8, 62.0)	40.0 (30.5, 54.5)	57.0 (45.0, 65.0)	**<0.01**
Gender, n(%)				0.99
Male	44 (45.8)	27 (45.8)	17 (46.0)	
Female	52 (54.2)	32 (54.2)	20 (54.1)	
Cancer, n(%)	11 (11.5)	4 (6.8)	7 (18.9)	0.14
RheD, n(%)	27 (28.1)	24 (40.7)	3 (8.1)	**<0.01**
Post-transplantation, n(%)	6 (6.3)	2 (3.4)	4 (10.8)	0.30
Time in hospital, M (Q_11_, Q_3_) (days)	14.5 (7.8, 26.5)	20.0 (12.0, 31.0)	7.0 (3.0, 14.0)	**<0.01**
Follow-up duration, M (Q_1_, Q_3_) (days)	55.0 (9.0, 623.8)	355.0 (85.0, 1, 627.5)	8.0 (3.0, 14.0)	**<0.01**
PLT, M (Q_1_, Q_3_) (10^9^/L)	10.0 (6.8, 27.5)	10.0 (8.0, 28.0)	9.0 (6.0, 27.0)	0.53
MPV, M (Q_1_, Q_3_) (fL)	9.6 (9.1, 10.3)	9.6 (9.1, 10.5)	9.6 (9.1, 10.2)	0.68
PDW, M (Q_1_, Q_3_) (fL)	10.8 (9.6, 13.9)	11.1 (9.7, 14.4)	10.0 (9.0, 10.8)	0.12
HGB, M (Q_1_, Q_3_) (g/L)	81.5 (66.0, 104.3)	85.0 (67.0, 105.5)	76.0 (60.0, 100.0)	0.42
RBC, M (Q_1_, Q_3_) (10^12^/L)	2.6 (2.0, 3.3)	2.7 (2.1, 3.4)	2.5 (2.0, 3.1)	0.44
MCV, M (Q_1_, Q_3_) (fL)	93.8 (85.4, 97.4)	93.9 (89.6, 98.3)	91.9 (84.0, 97.1)	0.21
Ret%, M (Q_1_, Q_3_) (%)	7.5 (3.6, 10.9)	7.6 (3.5, 12.3)	6.9 (3.6, 9.7)	0.92
Ret#, Mean ± SD (10^9^/L)	187.1 ± 121.1	192.4 ± 129.4	178.8 ± 106.5	0.61
WBC, M (Q_1_, Q_3_) (10^12^/L)	6.7 (5.1, 9.9)	6.2 (4.8, 10.2)	7.8 (5.5, 9.8)	0.18
NE, M (Q_1_, Q_3_) (10^9^/L)	4.9 (3.3, 7.0)	4.1 (3.1, 6.5)	5.8 (4.0, 7.4)	0.08
LY, M (Q_1_, Q_3_) (10^9^/L)	1.3 (0.8, 1.9)	1.19 (0.7, 1.6)	1.4 (1.0, 2.1)	0.11
CRP, Mean ± SD (mg/L)	8.0 (2.4, 21.4)	4.4 (1.9, 10.6)	21.4 (8.0, 44.6)	**<0.01**
PCT, M (Q_1_, Q_3_) (ng/mL)	0.1 (0.1, 0.3)	0.1 (0.1, 0.2)	0.3 (0.1, 0.5)	**0.01**
ALT, Mean ± SD (U/L)	28.0 (20.0, 42.0)	27.0 (18.0, 46.0)	29.0 (22.0, 42.0)	0.31
AST, M (Q_1_, Q_3_) (U/L)	50.0 (37.3, 78.8)	42.0 (29.0, 71.0)	62.0 (42.0, 87.0)	**0.01**
Cre, M (Q_1_, Q_3_) (μmol/L)	87.5 (71.8, 121.5)	80.0 (66.0, 108.5)	101.0 (81.0, 135.0)	**0.02**
LDH, M (Q_1_, Q_3_) (U/L)	1, 071.5 (594.3, 1, 697.3)	871.0 (530.0, 1, 401.0)	1, 522.0 (822.0, 2, 126.0)	**<0.01**
Tbil, Mean ± SD (μmol/L)	59.1 (30.8, 80.9)	54.8 (28.4, 76.7)	64.1 (43.0, 81.7)	0.12
Ibil, Mean ± SD (μmol/L)	39.7 (22.6, 59.2)	38.5 (18.0, 58.8)	42.3 (30.7, 63.6)	0.24
PT, M (Q_1_, Q_3_) (s)	12.2 (11.5, 13.4)	12.1 (11.4, 13.1)	12.6 (11.6, 14.3)	0.14
FIB, M (Q_1_, Q_3_) (mg/dL)	296.5 (242.8, 361.5)	300.0 (254.5, 356.0)	289.0 (220.0, 381.0)	0.80
APTT, M (Q_1_, Q_3_) (s)	29.6 (27.4, 33.1)	30.7 (28.1, 33.6)	28.5 (26.7, 31.3)	0.10
INR, M (Q_1_, Q_3_)	1.1 (1.0, 1.2)	1.1 (1.0, 1.2)	1.2 (1.1, 1.3)	0.08
FDP, Mean ± SD (mg/L)	7.7 (3.3, 15.6)	6.1 (3.0, 13.9)	9.8 (4.2, 25.3)	**0.04**
D-dimer, Mean ± SD (mg/L)	887.0 (495.0, 2, 022.0)	782.1 (376.8, 1, 305.0)	1, 322.0 (636.0, 2, 931.0)	**0.03**
LVEF, M (Q_1_, Q_3_) (%)	64.3 ± 11.8	66.9 ± 7.8	59.5 ± 16.2	**0.04**
Relapse, n(%)	8 (8.3)	8 (13.6)	0 (0.0)	0.05
Neurology, n(%)	86 (89.6)	52 (88.1)	34 (91.9)	0.81
Fever, n(%)	84 (87.5)	48 (81.4)	36 (97.3)	**0.04**
Bleeding, n(%)	73 (76.0)	44 (74.6)	29 (78.4)	0.67
Renal injury, n(%)	49 (51.0)	20 (33.9)	29 (78.4)	**<0.01**
MAHA, n(%)	94 (97.9)	58 (98.3)	36 (97.3)	1.00
Pentad, n(%)	47 (49.0)	19 (32.2)	28 (75.7)	**<0.01**
ADAMTS13%, n(%)	35 (36.5)	24 (40.7)	11 (29.7)	0.28
ADAMTS13I, n(%)	25 (26.0)	16 (27.1)	9 (24.3)	0.76

SD: standard deviation, M: Median, Q_1_: 1st Quartile, Q_3_: 3rd Quartile.

RheD, rheumatologic and autoimmune diseases; Post-transplantation, previous solid organ or hematopoietic stem cell transplantation; PLT, platelet count; HGB, hemoglobin; RBC, red blood cell count; MCV, mean corpuscular volume; Ret%, reticulocyte percentage; Ret#, reticulocyte absolute count; WBC, white blood cell count; NE, neutrophil count; LY, lymphocyte count; CRP, C-reactive protein; PCT, procalcitonin; ALT, alanine aminotransferase; AST, aspartate aminotransferase; Cre, serum creatinine; Tbil, total bilirubin; Ibil, indirect bilirubin; LDH, lactate dehydrogenase; PT, prothrombin time; FIB, fibrinogen; APTT, activated partial thromboplastin time; INR, international normalized ratio; FDP, fibrin degradation products; LVEF, left ventricular ejection fraction; Neurology, neurologic symptoms; MAHA, microangiopathic hemolytic anemia; Pentad, classic TTP pentad (thrombocytopenia, MAHA, neurologic symptoms, renal dysfunction, fever); ADAMTS13%, ADAMTS13 activity testing was performed and ADAMTS13 activity < 10%; ADAMTS13I, ADAMTS13 inhibitor testing was performed with a positive result.

Bold values indicate statistically significance (*P* < 0.05).

Among the variables, age, previous clinical history of rheumatologic and immunologic diseases, length of hospital stay, presence of renal injury, presence of the pentad of TTP, and follow-up time significantly differed between patients with mortality outcome and those without. The levels of C-reactive protein (CRP), procalcitonin (PCT), aspartate aminotransferase (AST), serum creatinine, LDH, fibrin degradation products (FDP), and D-dimer in patients who died were significantly greater than those in patients who survived during the follow-up period, whereas the left ventricular ejection fraction (LVEF) in patients who died outcome was significantly lower than that in the survival group. Fever, renal injury, and the classic pentad of TTP were more frequently observed in the mortality group.

Eight patients experienced relapse during the follow-up period, resulting in a cumulative incidence of 13.6% in the survival group. No relapse was observed in patients in the non-survival group. A comparison of the baseline characteristics and clinical features between patients who survived with and without relapse is presented in **Supplementary Table 1**. No significant difference was observed between the groups.

### 3.2 Treatment and treatment response

**Figure 2:**
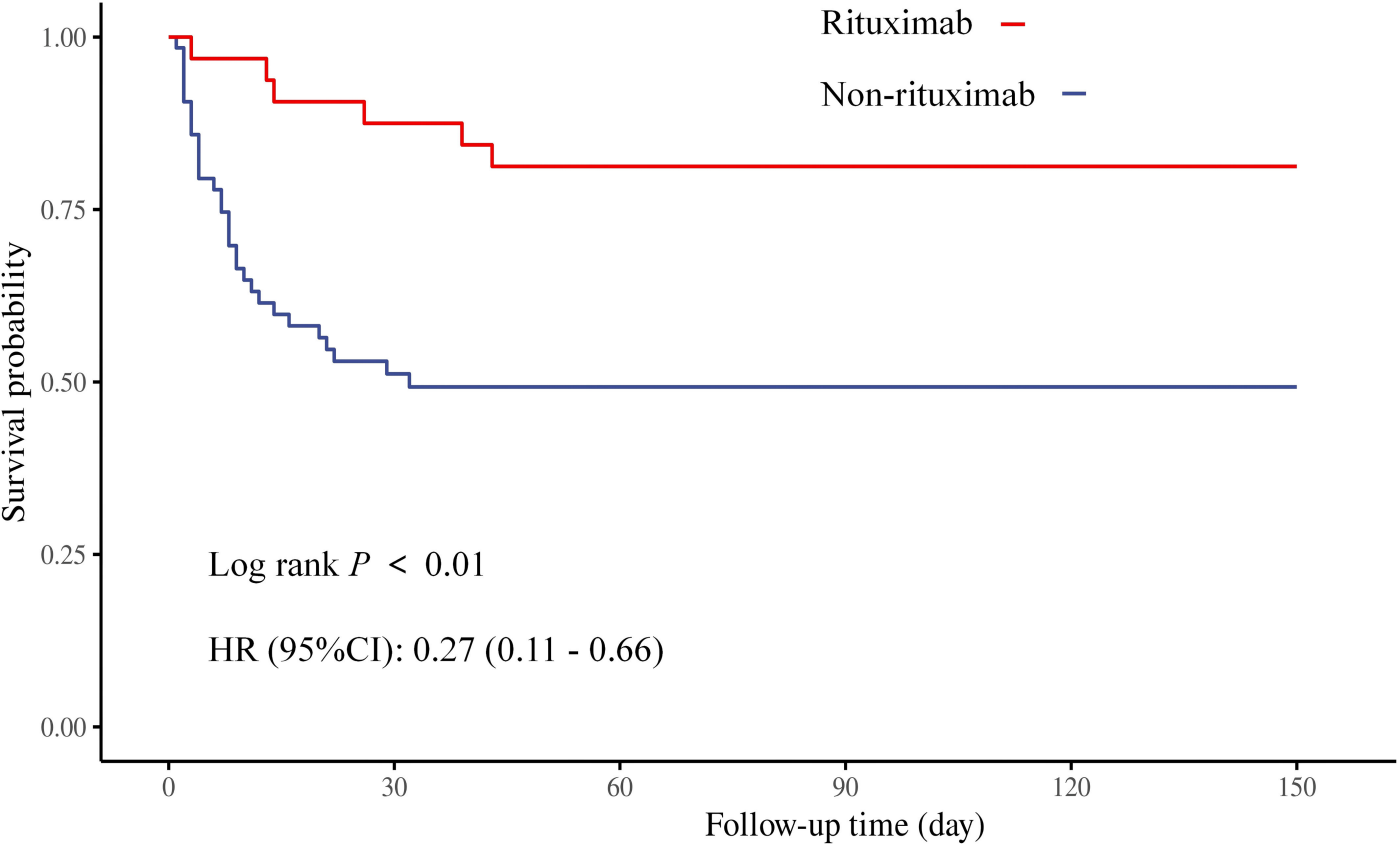
Survival curve of iTTP patients treated with or without rituximab.

The treatments and treatment response of the patients were compared on the basis of mortality or survival outcome, and the results are presented in **Table 2**. The response to treatment showed a significant difference between the groups. The survival curves of the different treatment methods and the analysis results of the log-rank test are displayed in **Figure 2** and **Supplementary Figures 1-3**. Although there was no significant difference, patients who received PE, corticosteroids, or corticosteroid pulse therapy treatment generally had better prognostic outcomes. The long-term survival outcomes of patients who received RTX treatment were significantly better than those of patients who did not. In addition, taking every seven-year period as a time frame, the comparison results in **Supplementary Table 2** reflect that the survival outcomes and clinical response of the patients tended to improve. The application of RTX also increased with the time period.

**Table 2:** The treatment and treatment response of patients in this study.

Variables	Overall (n = 96)	Survival (n = 59)	Death (n = 37)	*P*
Response, n(%)	39 (40.6)	33 (55.9)	6 (16.2)	**<0.01**
Platelet transfusion, n(%)	32 (33.3)	17 (28.8)	15 (40.5)	0.24
PE, n(%)	63 (65.6)	41 (69.5)	22 (59.5)	0.31
PEV, M (Q_1_, Q_3_)	9, 430.0 (6, 480.0, 13, 850.0)	9, 430.0 (7, 378.8, 14, 150.0)	9, 050.0 (5, 612.5, 13, 121.3)	0.31
PE times, M (Q_1_, Q_3_)	2.0 (0.8, 4.0)	2.0 (1.0, 4.0)	2.0 (0.0, 5.0)	0.92
Corticosteroids, n(%)	94 (97.9)	58 (98.3)	36 (97.3)	1.00
corticosteroid pulse therapy, n(%)	50 (52.1)	32 (54.2)	18 (48.7)	0.59
RTX, n(%)	32 (33.3)	26 (44.1)	6 (16.2)	**0.01**
Renal Replacement Therapy, n(%)	7 (7.3)	2 (3.4)	5 (13.5)	0.15

SD: standard deviation, M: Median, Q_1_: 1st Quartile, Q_3_: 3rd Quartile.

Response, clinical response; PE, plasma exchange; PEV, the total volume of plasma used in PE; PE times, the times of PE; RTX, rituximab.

Bold values indicate statistically significance (*P* < 0.05).

Univariate and multivariate logistic regression analyses were also performed to explore potential predictors of clinical response in first-episode iTTP patients as well. The results, which indicated that corticosteroid pulse therapy were related to clinical response, of univariate and multivariate logistic regression was presented in **Supplementary Table 3** and **Supplementary Figure 1**.

### 3.3 Predictors of prognostic outcomes

**Figure 3:**
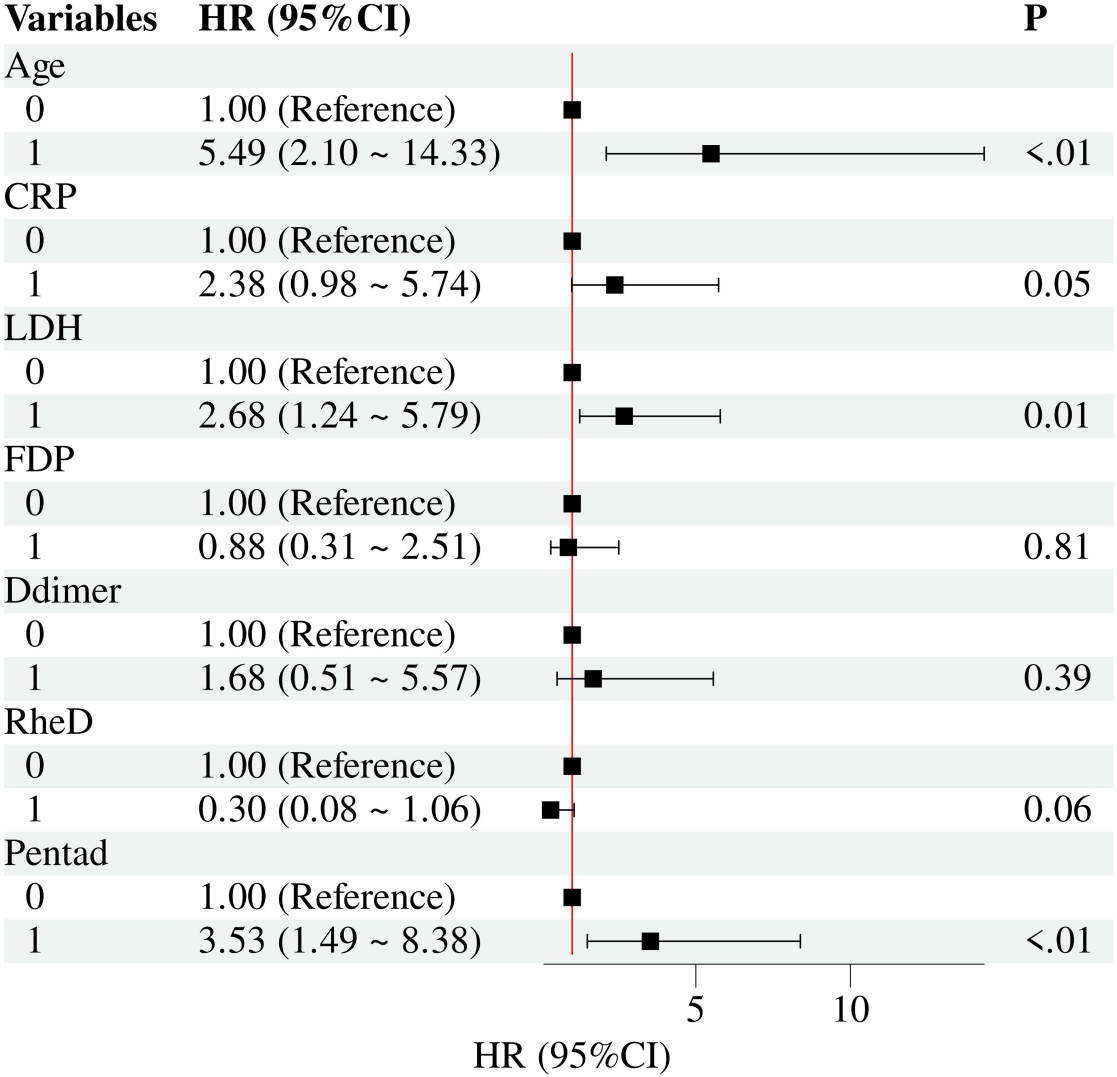
Forest plot of the multivariate Cox regression analysis results. Age, age over 45 years old; CRP, C-reactive protein over 14 mg/L; LDH, lactate dehydrogenase over 1500 U/L; FDP, fibrin degradation products over 7 mg/L; D-dimer, D-dimer over 1300 mg/L; RheD, rheumatologic and autoimmune diseases; Pentad, classic TTP pentad (thrombocytopenia, MAHA, neurologic symptoms, renal dysfunction, fever).

To identify predictors of mortality, univariate and multivariate Cox regression analyses were performed. Potential predictors were first screened via correlation analysis and univariate analysis. Age, CRP, LDH, FDP, D-dimer, history of rheumatologic and autoimmune diseases, and the presence of the pentad of TTP were identified with a *P* value < 0.1. We plotted receiver operating characteristic (ROC) curves for the continuous variables screened via univariate regression analysis, and determined their optimal cut-off values based on both data analysis results and clinical experience. The established cutoff values for the following continuous variables are as follows: age (45 years), LDH (1500 U/L), CRP (14 mg/L), D-dimer (1300 mg/L), and FDP (7 mg/L). These continuous variables were subsequently converted into binary variables and included in the multivariate Cox regression model. The results, as shown in **Table 3**, indicated that the presence of the pentad of TTP, advanced age and elevated LDH were independent predictors of mortality in our cohort. **Figure 3** shows the forest plot of the multivariate Cox regression analysis results. The predictors of relapse were also explored through Cox regression analyses, and no variables demonstrated statistically significant results in the multivariate regression analysis (**Supplementary Table 4**).

**Table 3:** Independent predictors of mortality in people with first-episode iTTP.

Variables	Univariate			Multivariate		
	HR	95% CI	*P*	HR	95% CI	*P*
Age	3.8	1.8 - 8.0	**<0.01**	5.5	2.1 - 14.3	**<0.01**
CRP	4.4	2.1 - 9.1	**<0.01**			
LDH	3.6	1.9 - 6.9	**<0.01**	2.7	1.2 - 5.8	0.01
FDP	1.9	1.0 - 3.8	0.05			
D-dimer	2.3	1.2- 4.4	**0.01**			
RheD	0.2	0.1 - 0.6	**<0.01**			
Pentad	4.6	2.2 - 9.8	**<0.01**	3.5	1.5 - 8.4	**<0.01**

HR, Hazard Ratio; CI, Confidence Interval; iTTP, immune thrombotic thrombocytopenic purpura; Age, age over 45 years old; CRP, C-reactive protein over 14 mg/L; LDH, lactate dehydrogenase over 1500 U/L; FDP, fibrin degradation products over 7 mg/L; D-dimer, D-dimer over 1300 mg/L; RheD, rheumatologic and autoimmune diseases; Pentad, classic TTP pentad (thrombocytopenia, MAHA, neurologic symptoms, renal dysfunction, fever).

Bold values indicate statistically significance (*P* < 0.05).

## 4 Discussion

ITTP is unquestionably an infrequent yet fatal thrombotic disorder. Timely and effective diagnosis and intervention are paramount for improving patient outcomes, particularly in comprehensive emergency departments and centers. To the best of our knowledge, this study is the first to systemically summarize the emergency experience in managing first-episode iTTP. The key findings of our study are as follows: (1) This study characterized the baseline demographics and clinical features of 96 patients with first-episode iTTP who presented to a single emergency center. (2) First-line treatments demonstrated a favorable effect on the prognosis of these patients and patients who received RTX exhibited significantly superior prognostic outcomes. (3) Multivariate analysis identified several independent predictors of mortality in first-episode iTTP patients, including the presence of the classic pentad of TTP symptoms, advanced age, elevated LDH levels, and the absence of a previous rheumatologic or autoimmune disease history. (4) Corticosteroid pulse therapy may enhance treatment responsiveness in patients with first-episode iTTP.

Our findings align with those of previous studies in many respects. For instance, we observed that the levels of LDH, serum creatinine, and D-dimer in the iTTP-related mortality group were significantly greater than those in the survival group, which is consistent with previous findings (12, 13). Elevated LDH levels are indicative of exacerbated hemolysis, while increased creatinine concentrations reflect impaired renal function. Additionally, elevated D-dimer levels signify increased fibrinolytic activity and the decreased LVEF shows a lower level of cardiac function. Collectively, these biomarker alterations and indicators underscore the severity of iTTP and its impact on multiple organ systems.

Regarding treatment response, the total rate of clinical response reported in our study is 40.625%. This lower treatment response rate does not imply poor treatment efficacy, given that all patients in the survival group included in this study were discharged after receiving treatment and exhibited significantly improved clinical manifestations. The possible reason is that some enrolled patients were discharged early from our emergency center and lacked continuous laboratory follow-up results, which led to their failure to meet some of the criteria for a clinical response (sustained platelet count ≥ 150 × 10^9^/L and LDH < 1.5 times the upper limit of normal).

We found that RTX significantly improved survival outcomes in first-episode iTTP patients, providing additional evidence to support the current clinical consensus advocating for early RTX initiation in iTTP management (1, 7, 14). Notably, the survival benefit of RTX combined with corticosteroids in our cohort (HR = 0.27, 95% CI: 0.11-0.66) exceeds the effect sizes reported in a retrospective study conducted in the United States (HR = 0.37, 95% CI: 0.18-0.73) (15). This disparity may be attributable to the emergency department-initiated RTX administration protocol in our study, which contrasts with delayed outpatient treatment strategies. Although statistical significance was not achieved, patients receiving PE and corticosteroids tended to exhibit favorable prognostic outcomes, aligning with current clinical guidelines that recommend PE and corticosteroids as first-line therapies (1, 7). The absence of significant results for PE treatment might be explained by the limited plasma availability in our emergency center, which could have led to suboptimal PE volumes and consequently compromised treatment efficacy (12). Considering that most of the patients included in this study (94 patients, 97.9%) received corticosteroid treatment, the limited number of patients not receiving corticosteroids may be the reason for the negative statistical results. Additionally, our analysis identified corticosteroid pulse therapy as a potential independent predictor of clinical response. While the role of corticosteroid pulse therapy in iTTP management remains controversial (2, 16), our findings underscore the importance of early and adequate corticosteroid administration, in line with existing therapeutic recommendations (17).

In terms of predictors of mortality, advanced age and elevated LDH levels have been previously identified as significant mortality predictors in prior studies related to the iTTP (13, 18). In elderly patients, baseline organ function is generally compromised relative to that of younger individuals, with the cardiovascular system being particularly vulnerable. This age-related decline is inherently linked to physiological degenerative changes in vascular structure and function, such as endothelial dysfunction, arterial stiffening, and reduced vascular compliance, all of which exacerbate the disease burden in iTTP (19). Beyond this, elevated LDH levels serve as a dual biomarker in iTTP: they not only indicate severe microangiopathic hemolysis but also signal widespread multiorgan involvement, encompassing critical organs such as the brain, kidneys, heart, gastrointestinal tract, and liver (20, 21). These two factors collectively reflect the potential impact of poor organ function on iTTP prognosis. Additionally, we identified TTP pentad as a mortality predictor. Although current clinical consensus acknowledges that the classic TTP pentad (thrombocytopenia, microangiopathic hemolytic anemia, neurological symptoms, renal dysfunction, and fever) is rarely observed in its complete form in real-world practice (2, 22), its partial or full manifestation is indicative of an aggressive iTTP phenotype characterized by diffuse microvascular thrombosis and severe multiorgan damage. This triggers rapid clinical deterioration and confers a substantially elevated risk of adverse outcomes. This finding underscores the need for intensified multiorgan monitoring, particularly of renal function, in iTTP management. However, there were no significant predictors of relapse identified in this study, which might be attributed to the relatively low relapse rate among the enrolled patients (8 cases, 13.6% in the survival group) (23, 24). This result stems from both our optimized management of first-episode iTTP and potential biases in follow-up data. Notably, 38.5% of patients died during follow-up and were excluded from relapse analysis, so assessments were restricted to the non-death group. Moreover, prompt emergency interventions at our center improved patient outcomes and lowered relapse risk. As an emergency center, we used a mixed telephone-outpatient follow-up strategy. However, some patients might transition to outside specialized care, potentially introducing bias.

The insights derived from this study offer several clinical implications. First, we provide valuable real-world evidence characterizing the baseline demographics and clinical features of first-episode iTTP patients presenting to an emergency center. Although existing mortality risk prediction models for iTTP can identify high-risk patients to a certain degree (25, 26), they still have limitations, including limited prediction accuracy and insufficient external validation of the models (26). The identified prognostic predictors of first-episode iTTP in this study (age, LDH, and pentad symptoms) provide a risk stratification framework for emergency clinicians, enabling more precise triage, guiding targeted multiorgan function monitoring and decisions regarding intensive care unit admission or therapeutic escalation.

Our findings also highlight the critical role of early RTX initiation in acute iTTP management. The trend of improved survival over time (mortality rate decreasing from 54.5% in 2004–2010 to 30.8% in 2018-2024) further validates the clinical impact of evolving management, particularly the increased adoption of RTX, which rose from 0% to 51.9% during the study period. Moreover, the association between corticosteroid pulse therapy and clinical response (OR = 2.8, *P* = 0.04) supports the utility of high-dose initial corticosteroids in emergency settings. This approach may expedite platelet recovery and improve organ function, serving as a critical bridge therapy in resource-constrained centers where PE or RTX initiation is delayed.

This study has several limitations that should be acknowledged. First, the single-center design, which is based on a tertiary referral emergency center, may limit generalizability due to regional variations in disease epidemiology and healthcare access. Comprehensive data on ADAMTS13 activity/antibody detection, along with data pertaining to the efficacy and safety of caplacizumab, are unavailable in our study cohort. Additionally, both the retrospective nature of the analysis and subsequent follow-up procedures may introduce bias, potentially leading to an overestimation of treatment efficacy or underestimation of mortality. Future studies should focus on externally validating our conclusions and constructing predictive models in larger, multi-center cohorts. Incorporating novel predictors or biomarkers (e.g., ADAMTS13 activity kinetics, caplacizumab administration, and autoantibody specificity) may improve the predictive performance of risk stratification tools. Finally, developing AI-driven predictive models and decision support systems that integrate real-time laboratory data and clinical features could optimize emergency treatment algorithms and reduce delays in therapy—a critical step toward improving first-episode iTTP outcomes in the emergency management.

## 5 Conclusion

In conclusion, this study provides valuable insights into the treatment and independent predictors of the prognostic outcome of first-episode iTTP from the perspective of an emergency center. However, further research is needed to overcome the limitations of this study and to develop more effective and personalized treatment approaches for this life-threatening thrombotic disorder.

## Data Availability

The raw data supporting the conclusions of this article will be made available by the authors, without undue reservation.

## Supplementary material

Supplementary Table 1 Baseline and clinical characteristics of iTTP patients in survival.

Supplementary Table 2 The summary comparison of every seven-year period.

Supplementary Table 3 Independent predictors of clinical response of first-episode iTTP.

Supplementary Figure 1 Forest plot of the multivariate logistic regression analysis results.

Supplementary Figure 2 Survival curve of iTTP patients treated with or without plasma exchange.

Supplementary Figure 3 Survival curve of iTTP patients treated with or without corticosteriods.

Supplementary Figure 4 Survival curve of iTTP patients treated with or without corticosteriods pulse therapy.

Supplementary Figure 5 ROC curve of age as a predictor of mortality in patients with first-episode iTTP.

Supplementary Figure 6 ROC curve of C-reactive protein as a predictor of mortality in patients with first-episode iTTP.

Supplementary Figure 7 ROC curve of D-dimer protein as a predictor of mortality in patients with first-episode iTTP.

Supplementary Figure 8 ROC curve of lactate dehydrogenase as a predictor of mortality in patients with first-episode iTTP

Supplementary Figure 9 ROC curve of fibrin degradation products as a predictor of mortality in patients with first-episode iTTP
