# Supplementary material for: Experience in emergency management of first-episode immune thrombotic thrombocytopenic purpura over the past 21 years: a single-center retrospective study
Source: Front Immunol. 2026 Jan 14;16:1645558. doi: 10.3389/fimmu.2025.1645558 (PMC12847437; doi:10.3389/fimmu.2025.1645558)
Supplement: Supplementary Table 1 — Baseline and clinical characteristics of iTTP patients in survival. [file Table1.docx]

Supplementary Table 1. Baseline and clinical characteristics of iTTP patients in survival

| Variables | Overall (n = 59) | Non-relapse (n = 51) | Relapse (n = 8) | *P* |
| --- | --- | --- | --- | --- |
|  |  |  |  |  |
| Age, M (Q₁, Q₃) (years) | 40.0 (30.5, 54.5) | 40.0 (29.5, 53.0) | 40.0 (32.8, 60.5) | 0.45 |
| Gender, n(%) |  |  |  | 0.38 |
| Male | 27 (45.8) | 25 (49.0) | 2 (25.0) |  |
| Female | 32 (54.2) | 26 (51.0) | 6 (75.0) |  |
| Cancer, n(%) | 4 (6.8) | 4 (7.8) | 0 (0.0) | 1.00 |
| RheD, n(%) | 24 (40.7) | 22 (43.1) | 2 (25.0) | 0.56 |
| Post-Transplantation, n(%) | 2 (3.4) | 2 (3.9) | 0 (0.0) | 1.00 |
| Time in hospital, M (Q₁, Q₃) (days) | 20.0 (12.0, 31.0) | 20.0 (12.5, 31.0) | 15.5 (12.0, 25.8) | 0.64 |
| Follow-up duration, Mean ± SD (days) | 973.0 ± 1,225.6 | 888.2 ± 1,195.7 | 1,513.9 ± 1,359.3 | 0.18 |
| PLT, M (Q₁, Q₃) (10^9^/L) | 10.0 (8.0, 28.0) | 10.0 (8.0, 43.5) | 11.0 (3.0, 14.3) | 0.25 |
| MPV, M (Q₁, Q₃) (fL) | 9.7 (9.3, 10.6) | 9.6 (9.0, 10.5) | 10.2 (9.7, 11.6) | 0.27 |
| PDW, M (Q₁, Q₃) (fL) | 10.9 (9.6, 14.4) | 11.0 (9.6, 15.4) | 10.5 (9.9, 12.3) | 0.69 |
| HGB, M (Q₁, Q₃) (g/L) | 85.0 (67.0, 105.5) | 85.0 (68.0, 107.0) | 81.0 (65.8, 91.5) | 0.69 |
| RBC, M (Q₁, Q₃) (10^12^/L) | 2.7 (2.1, 3.4) | 2.7 (2.2, 3.4) | 2.5 (2.0, 2.8) | 0.52 |
| MCV, M (Q₁, Q₃) (fL) | 93.9 (89.6, 98.3) | 93.2 (89.4, 99.3) | 95.7 (92.7, 96.4) | 0.77 |
| Ret%, M (Q₁, Q₃) (%) | 7.6 (3.5, 12.3) | 5.4 (3.1, 12.3) | 9.73 (8.4, 11.0) | 0.13 |
| Ret#, Mean ± SD (10^9^/L) | 192.4 ± 129.4 | 177.5 ± 123.6 | 272.5 ± 138.6 | 0.06 |
| WBC, M (Q₁, Q₃) (10^12^/L) | 6.2 (4.8, 10.2) | 6.3 (4.8, 10.2) | 5.7 (5.2, 9.6) | 0.96 |
| NE, M (Q₁, Q₃) (10^9^/L) | 4.1 (3.1, 6.5) | 4.3 (3.2, 6.0) | 3.2 (2.9, 7.4) | 0.73 |
| LY, M (Q₁, Q₃) (10^9^/L) | 1.2 (0.7, 1.6) | 1.2 (0.7, 1.6) | 1.2 (0.6, 2.0) | 0.98 |
| CRP, Mean ± SD (mg/L) | 14.5 ± 32.5 | 15.9 ± 34.4 | 3.4 ± 2.8 | 0.38 |
| PCT, M (Q₁, Q₃) (ng/mL) | 0.1 (0.1, 0.2) | 0.2 (0.1, 0.2) | 0.2 (0.2, 0.2) | 0.60 |
| ALT, Mean ± SD (U/L) | 44.0 ± 94.3 | 44.3 ± 100.6 | 42.1 ± 19.9 | 0.96 |
| AST, M (Q₁, Q₃) (U/L) | 42.0 (29.0, 71.0) | 42.0 (27.5, 72.5) | 48.0 (42.0, 56.0) | 0.51 |
| Cre, M (Q₁, Q₃) (μmol/L) | 80.0 (66.0, 108.5) | 83.0 (69.0, 115.0) | 66.5 (58.0, 82.5) | 0.09 |
| LDH, M (Q₁, Q₃) (U/L) | 871.0 (530.0, 1,401.0) | 832.0 (530.0, 1,350.5) | 1,046.5 (601.0, 1,463.0) | 0.67 |
| Tbil, Mean ± SD (μmol/L) | 61.3 ± 54.5 | 59.9 ± 56.9 | 69.8 ± 37.7 | 0.64 |
| Ibil, Mean ± SD (μmol/L) | 43.6 ± 39.6 | 42.4 ± 41.0 | 51.2 ± 30.8 | 0.57 |
| PT, M (Q₁, Q₃) (s) | 12.1 (11.4, 13.1) | 12.1 (11.4, 13.1) | 12.2 (11.8, 12.8) | 0.81 |
| FIB, M (Q₁, Q₃) (mg/dL) | 300.0 (254.5, 356.0) | 287.0 (246.5, 356.0) | 330.0 (306.0, 357.5) | 0.20 |
| APTT, M (Q₁, Q₃) (s) | 30.7 (28.1, 33.6) | 31.1 (28.5, 34.1) | 29.5 (26.7, 32.2) | 0.37 |
| INR, M (Q₁, Q₃) | 1.1 (1.0, 1.2) | 1.1 (1.0, 1.2) | 1.1 (1.1, 1.2) | 0.86 |
| FDP, Mean ± SD (mg/L) | 12.1 ± 16.7 | 12.5 ± 17.2 | 9.9 ± 14.1 | 0.68 |
| D-dimer, Mean ± SD (mg/L) | 1,514.2 ± 2,082.2 | 1,540.5 ± 2,113.5 | 1,350.3 ± 2,000.4 | 0.81 |
| LVEF, M (Q₁, Q₃) (%) | 66.1 (61.5, 73.3) | 66.1 (61.5, 73.3) | 65.0 (61.0, 72.5) | 0.93 |
| Response, n(%) | 33 (55.9) | 27 (52.9) | 6 (75.0) | 0.43 |
| Neurology, n(%) | 52 (88.1) | 44 (86.3) | 8 (100.0) | 1.00 |
| Fever, n(%) | 48 (81.4) | 42 (82.4) | 6 (75.0) | 0.99 |
| Bleeding, n(%) | 44 (74.6) | 36 (70.6) | 8 (100.0) | 0.18 |
| Renal injury, n(%) | 20 (33.9) | 20 (39.2) | 0 (0.0) | 0.08 |
| MAHA, n(%) | 58 (98.3) | 50 (98.0) | 8 (100.0) | 1.00 |
| Pentad, n(%) | 19 (32.2) | 19 (37.3) | 0 (0.0) | 0.09 |
| ADAMTS13%, n(%) | 24 (40.7) | 19 (37.3) | 5 (62.5) | 0.34 |
| ADAMTS13I, n(%) | 16 (27.1) | 13 (25.5) | 3 (37.5) | 0.78 |
| PE, n(%) | 41 (69.5) | 34 (66.7) | 7 (87.5) | 0.44 |
| corticosteroids, n(%) | 58 (98.3) | 50 (98.0) | 8 (100.0) | 1.00 |
| corticosteroid pulse therapy, n(%) | 32 (54.2) | 28 (54.9) | 4 (50.0) | 0.58 |
| RTX, n(%) | 26 (44.1) | 23 (45.1) | 3 (37.5) | 0.98 |
| SD: standard deviation, M: Median, Q₁: 1st Quartile, Q₃: 3rd Quartile | | | | |

Abbreviations: iTTP, immune thrombotic thrombocytopenic purpura; RheD: rheumatologic and autoimmune diseases; Post-transplantation, previous solid organ or hematopoietic stem cell transplantation; PLT, platelet count; HGB, hemoglobin; RBC, red blood cell count; MCV, mean corpuscular volume; Ret%, reticulocyte percentage; Ret#, reticulocyte absolute count; WBC, white blood cell count; NE, neutrophil count; LY, lymphocyte count; CRP, C-reactive protein; PCT, procalcitonin; ALT, alanine aminotransferase; AST, aspartate aminotransferase; Cre, serum creatinine; Tbil, total bilirubin; Ibil, indirect bilirubin; LDH, lactate dehydrogenase; PT, prothrombin time; FIB, fibrinogen; APTT, activated partial thromboplastin time; INR, international normalized ratio; FDP, fibrin degradation products; LVEF, left ventricular ejection fraction; Neurology, neurologic symptoms; MAHA, microangiopathic hemolytic anemia; Pentad, classic TTP pentad (thrombocytopenia, MAHA, neurologic symptoms, renal dysfunction, fever); ADAMTS13%, ADAMTS13 activity testing was performed and ADAMTS13 activity < 10%; ADAMTS13I, ADAMTS13 inhibitor testing was performed with a positive result, PE, plasma exchange; RTX, rituximab.

Bold values indicate statistically significance (*P* < 0.05).
