# Supplementary material for: Experience in emergency management of first-episode immune thrombotic thrombocytopenic purpura over the past 21 years: a single-center retrospective study
Source: Front Immunol. 2026 Jan 14;16:1645558. doi: 10.3389/fimmu.2025.1645558 (PMC12847437; doi:10.3389/fimmu.2025.1645558)
Supplement: Supplementary Table 2 — The summary comparison of every seven-year period. [file Table2.docx]

Supplementary Table 2. The summary comparison of every seven-year period

| Variables | Overall(n=96) | 2004-2010(n=11) | 2011-2017(n=33) | 2018-2024(n=52) | *P* |
| --- | --- | --- | --- | --- | --- |
| Female (%) | 52 (54.2) | 8 (72.7) | 18 (54.5) | 26 (50.0) | 0.41 |
| age (mean (SD)) (years) | 47.0 (17.1) | 46.6 (12.7) | 46.7 (14.7) | 47.3 (19.5) | 0.99 |
| Time in hospital (mean (SD)) (days) | 18.0 (13.1) | 21.4 (14.2) | 16.8 (13.5) | 18.1 (12.7) | 0.60 |
| Follow-up duration(mean (SD)) (days) | 602.4 (1,067.0) | 1,039.3 (1,584.2) | 751.4 (1,365.9) | 415.4 (605.6) | 0.13 |
| Relapse (%) | 8 (8.3) | 2 (18.2) | 1 (3.0) | 5 (9.6) | 0.21 |
| Death (%) | 37 (38.5) | 6 (54.5) | 15 (45.5) | 16 (30.8) | 0.21 |
| Response(%) | 39 (40.6) | 1 (9.1) | 18 (54.5) | 20 (38.5) | **0.02** |
| Pentad (%) | 47 (49.0) | 6 (54.5) | 17 (51.5) | 24 (46.2) | 0.84 |
| PE (%) | 63 (65.6) | 9 (81.8) | 20 (60.6) | 34 (65.4) | 0.48 |
| Corticosteroids (%) | 94 (97.9) | 11 (100.0) | 32 (97.0) | 51 (98.1) | 1.00 |
| Corticosteroid pulse therapy(%) | 50 (52.1) | 4 (36.4) | 23 (69.7) | 23 (44.2) | 0.04 |
| RTX(%) | 32 (33.3) | 0 (0.0) | 5 (15.2) | 27 (51.9) | **<0.01** |
| SD: standard deviation, M: Median, Q₁: 1st Quartile, Q₃: 3rd Quartile | | | | | |

Abbreviations: Response, clinical response; Pentad, classic TTP pentad (thrombocytopenia, MAHA, neurologic symptoms, renal dysfunction, fever); PE, plasma exchange; RTX, rituximab.

Bold values indicate statistically significance (*P* < 0.05).
