# Supplementary material for: Experience in emergency management of first-episode immune thrombotic thrombocytopenic purpura over the past 21 years: a single-center retrospective study
Source: Front Immunol. 2026 Jan 14;16:1645558. doi: 10.3389/fimmu.2025.1645558 (PMC12847437; doi:10.3389/fimmu.2025.1645558)
Supplement: Supplementary Table 3 — Independent predictors of clinical response of first-episode iTTP. [file Table3.docx]

Supplementary Table 3**.** Independent predictors of clinical response of first-episode iTTP

| Variables | Univariate analysis | | | Multivariate analysis | | |
| --- | --- | --- | --- | --- | --- | --- |
|  | OR | 95% CI | *P* | OR | 95% CI | *P* |
| CRP | 0.98 | 0.96 - 0.99 | **0.05** |  |  |  |
| PLT | 0.99 | 0.98 - 1.00 | 0.08 |  |  |  |
| Pentad | 0.41 | 0.18 - 0.94 | **0.04** |  |  |  |
| Corticosteroid pulse therapy | 4.05 | 1.68 - 9.74 | **<0.01** | 2.82 | 1.05 - 7.55 | **0.04** |
| OR: Odds Ratio, CI: Confidence Interval | | | | | | |

Abbreviations: CRP, C-reactive protein; PLT, platelet count; Pentad, classic TTP pentad (thrombocytopenia, MAHA, neurologic symptoms, renal dysfunction, fever).

Bold values indicate statistically significance (*P* < 0.05).
