# Supplementary material for: Experience in emergency management of first-episode immune thrombotic thrombocytopenic purpura over the past 21 years: a single-center retrospective study
Source: Front Immunol. 2026 Jan 14;16:1645558. doi: 10.3389/fimmu.2025.1645558 (PMC12847437; doi:10.3389/fimmu.2025.1645558)
Supplement: Supplementary file 4 [file Table4.docx]

Supplementary Table 4. Independent predictors of relapse in people with first-episode iTTP

| Variables | Univariate analysis | | | Multivariate analysis | | |
| --- | --- | --- | --- | --- | --- | --- |
|  | HR | 95% CI | *P* | HR | 95% CI | *P* |
| Ret% | 1.1 | 1.0 - 1.2 | **0.04** | 0.9 | 0.6 - 1.2 | 0.34 |
| Ret# | 483.0 | 3.7 - 63,107.5 | **0.01** | 624,663.2 | 0.1 - 168,351,017,266,829.0 | 0.18 |
| Cre | 1.0 | 0.9 - 1.0 | 0.08 | 1.0 | 0.9 - 1.0 | 0.28 |
| HR: Hazard Ratio, CI: Confidence Interval | | | | | | |

Abbreviations: Ret%, reticulocyte percentage; Ret#, reticulocyte absolute count; Cre, serum creatinine.

Bold values indicate statistically significance (*P* < 0.05).
