# Supplementary figures and images for: Experience in emergency management of first-episode immune thrombotic thrombocytopenic purpura over the past 21 years: a single-center retrospective study
Source: Front Immunol. 2026 Jan 14;16:1645558. doi: 10.3389/fimmu.2025.1645558 (PMC12847437; doi:10.3389/fimmu.2025.1645558)

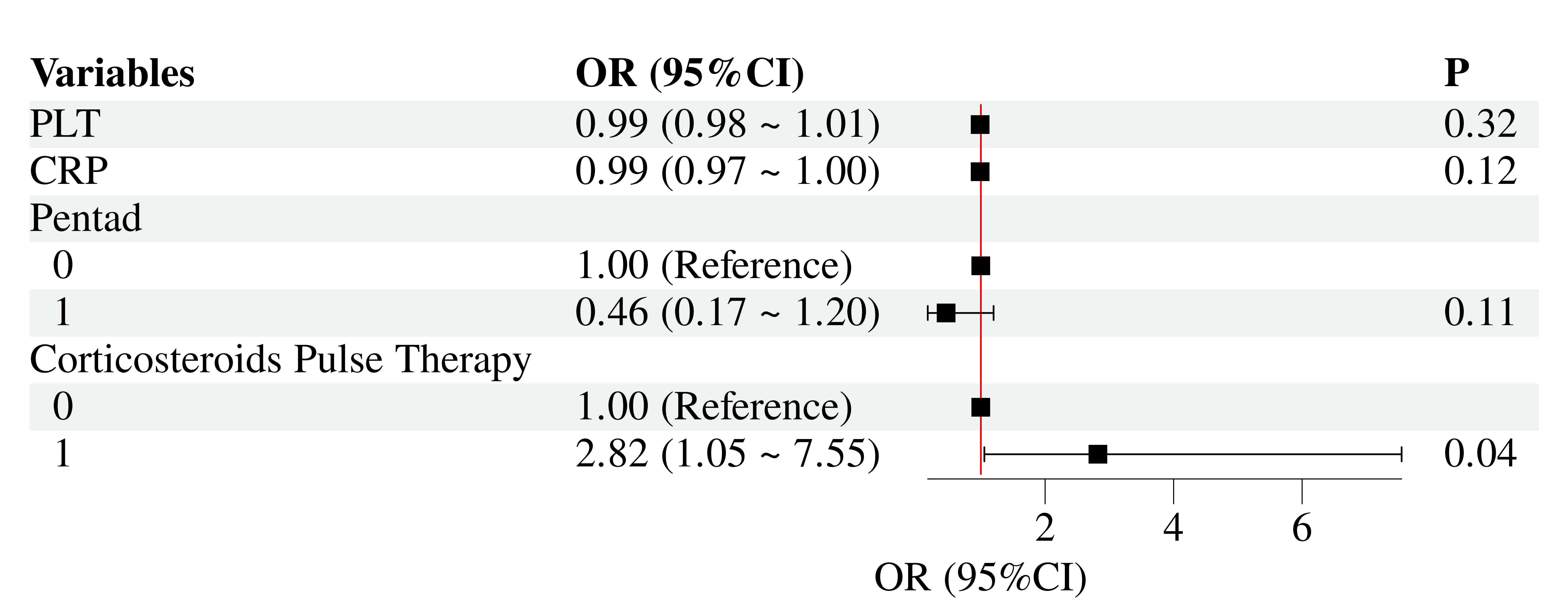

Supplement: Supplementary Figure 1 — Forest plot of the multivariate logistic regression analysis results. [file Image1.jpeg]

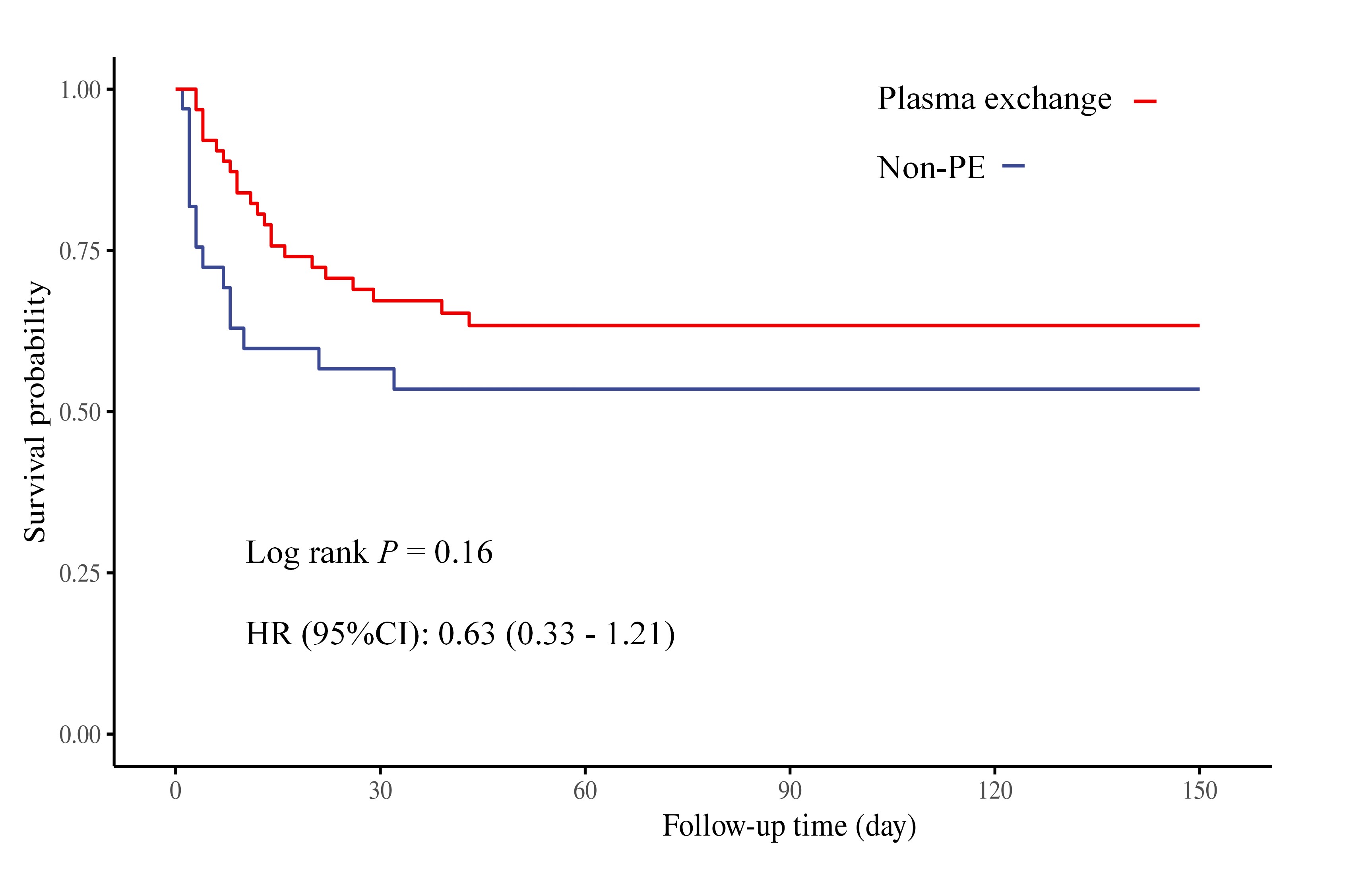

Supplement: Supplementary Figure 2 — Survival curve of iTTP patients treated with or without plasma exchange. [file Image2.jpeg]

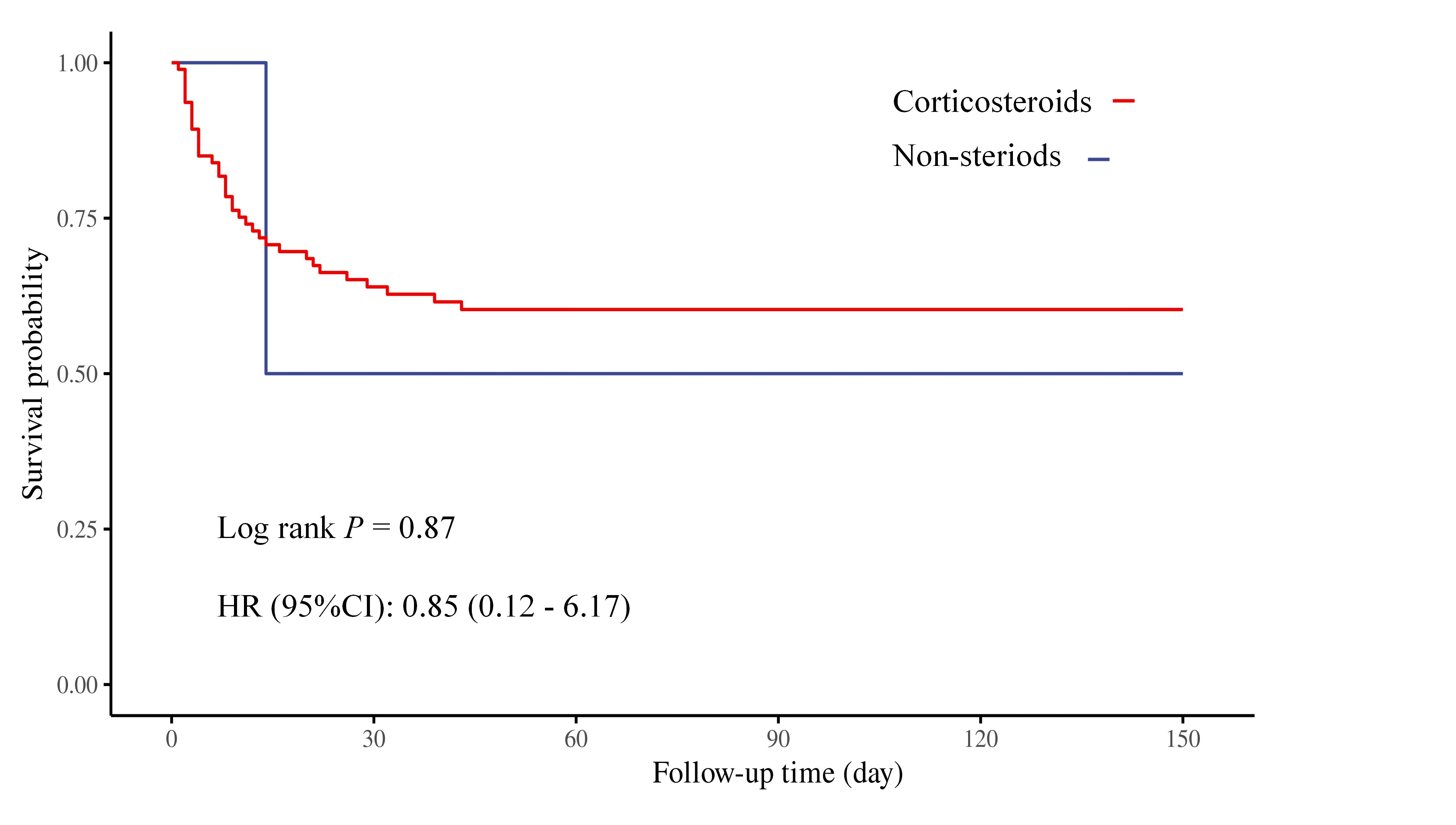

Supplement: Supplementary Figure 3 — Survival curve of iTTP patients treated with or without corticosteriods. [file Image3.jpeg]

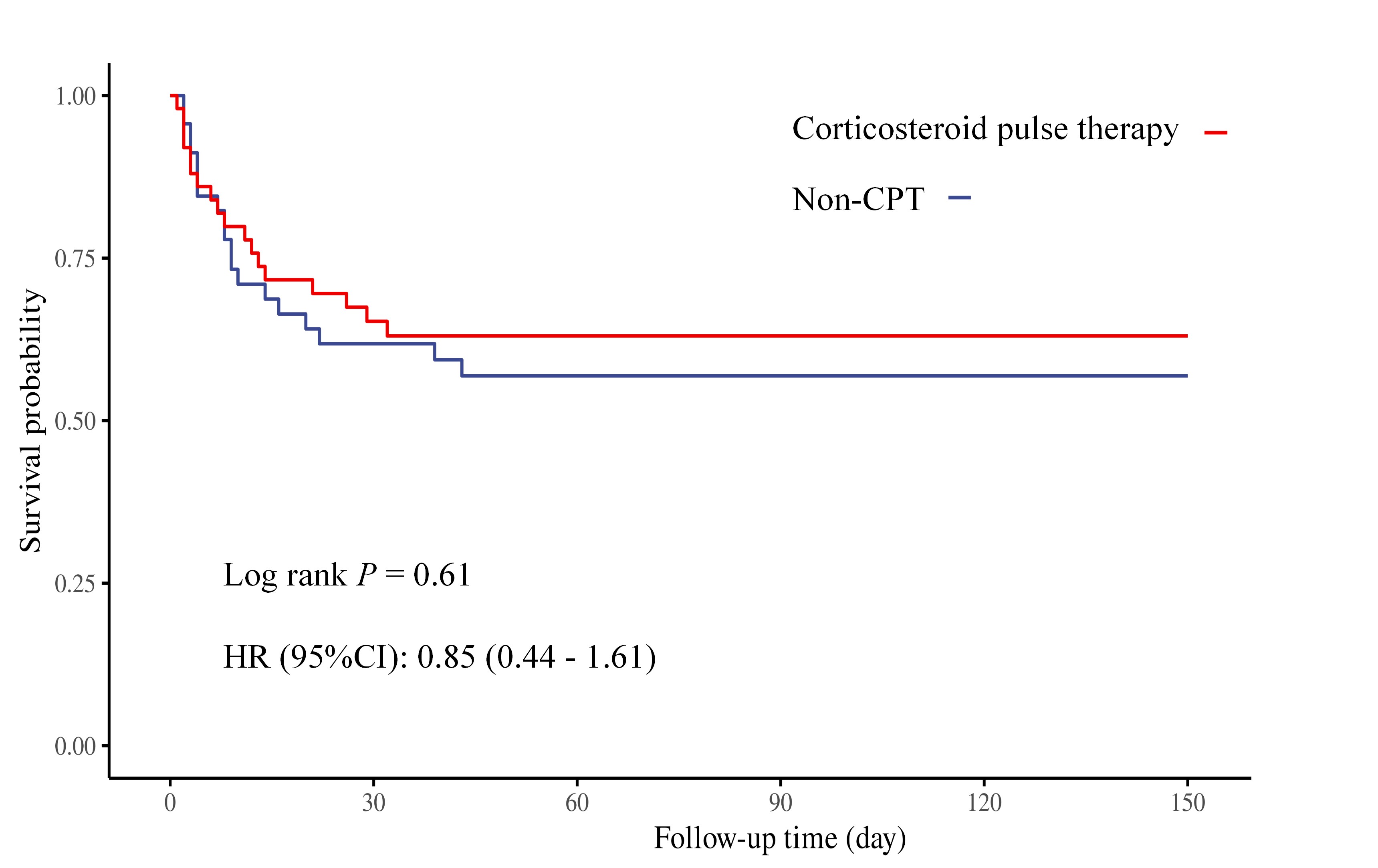

Supplement: Supplementary Figure 4 — Survival curve of iTTP patients treated with or without corticosteriods pulse therapy. [file Image4.jpeg]

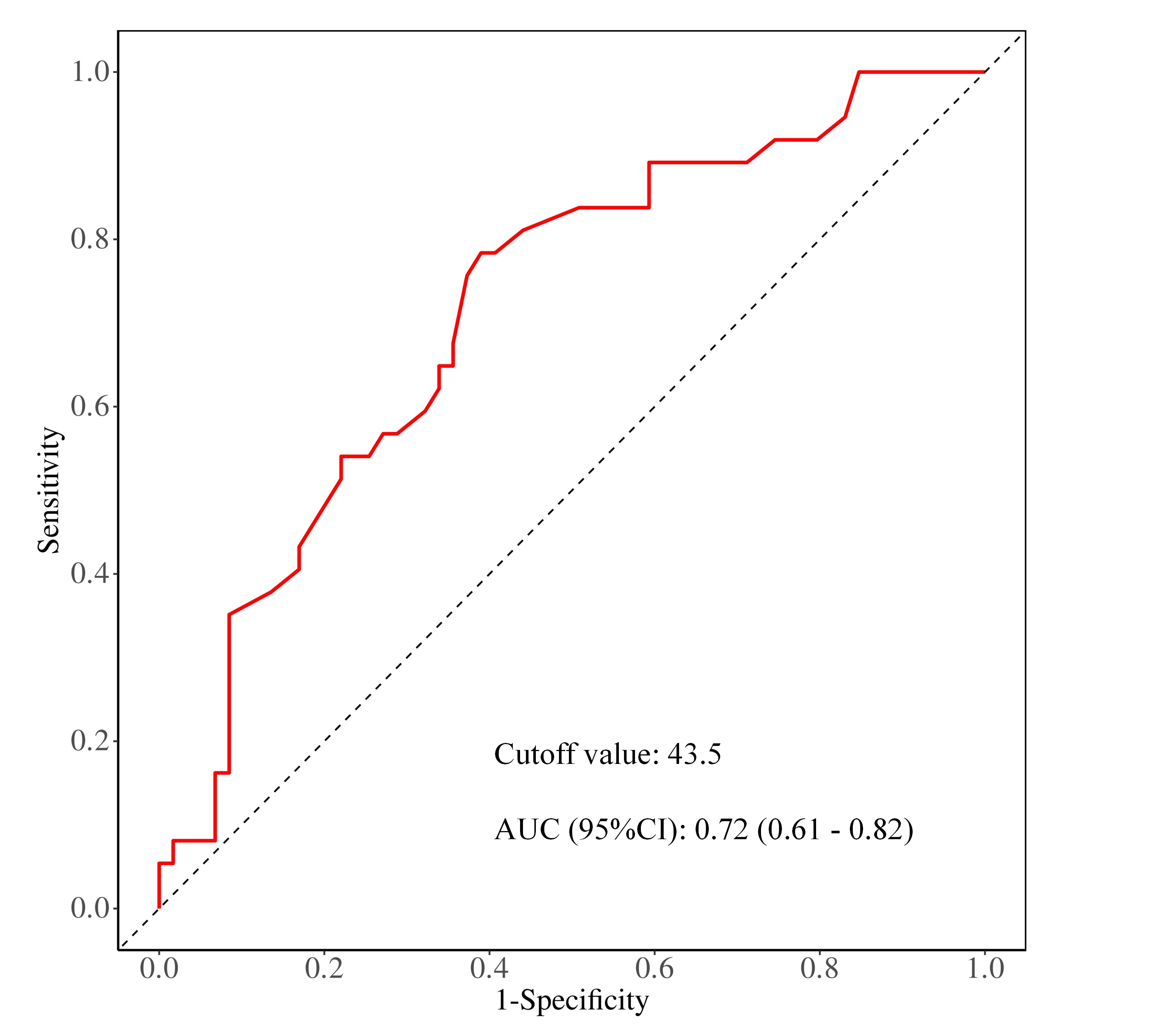

Supplement: Supplementary Figure 5 — ROC curve of age as a predictor of mortality in patients with first-episode iTTP. [file Image5.jpeg]

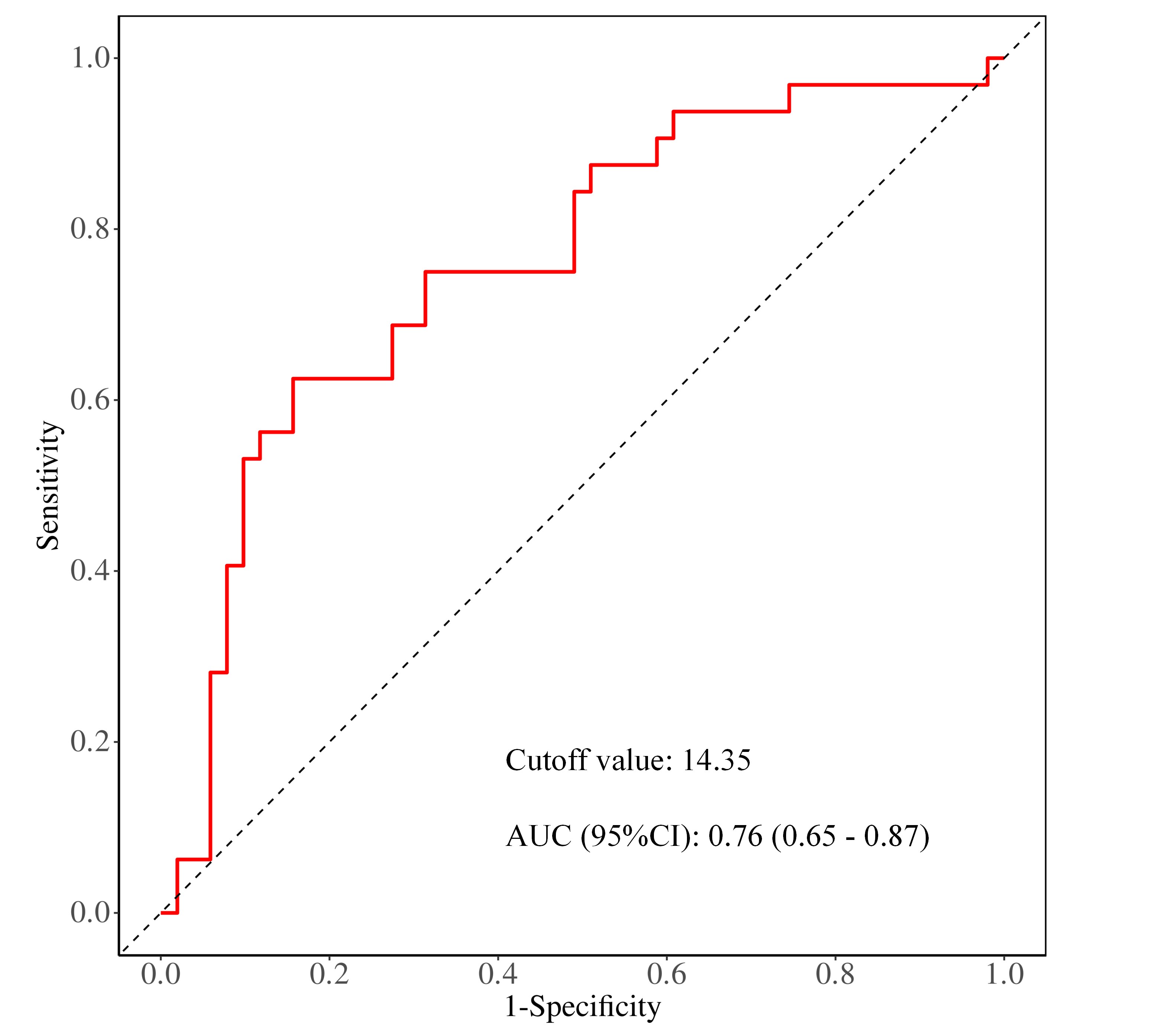

Supplement: Supplementary Figure 6 — ROC curve of C-reactive protein as a predictor of mortality in patients with first-episode iTTP. [file Image6.jpeg]

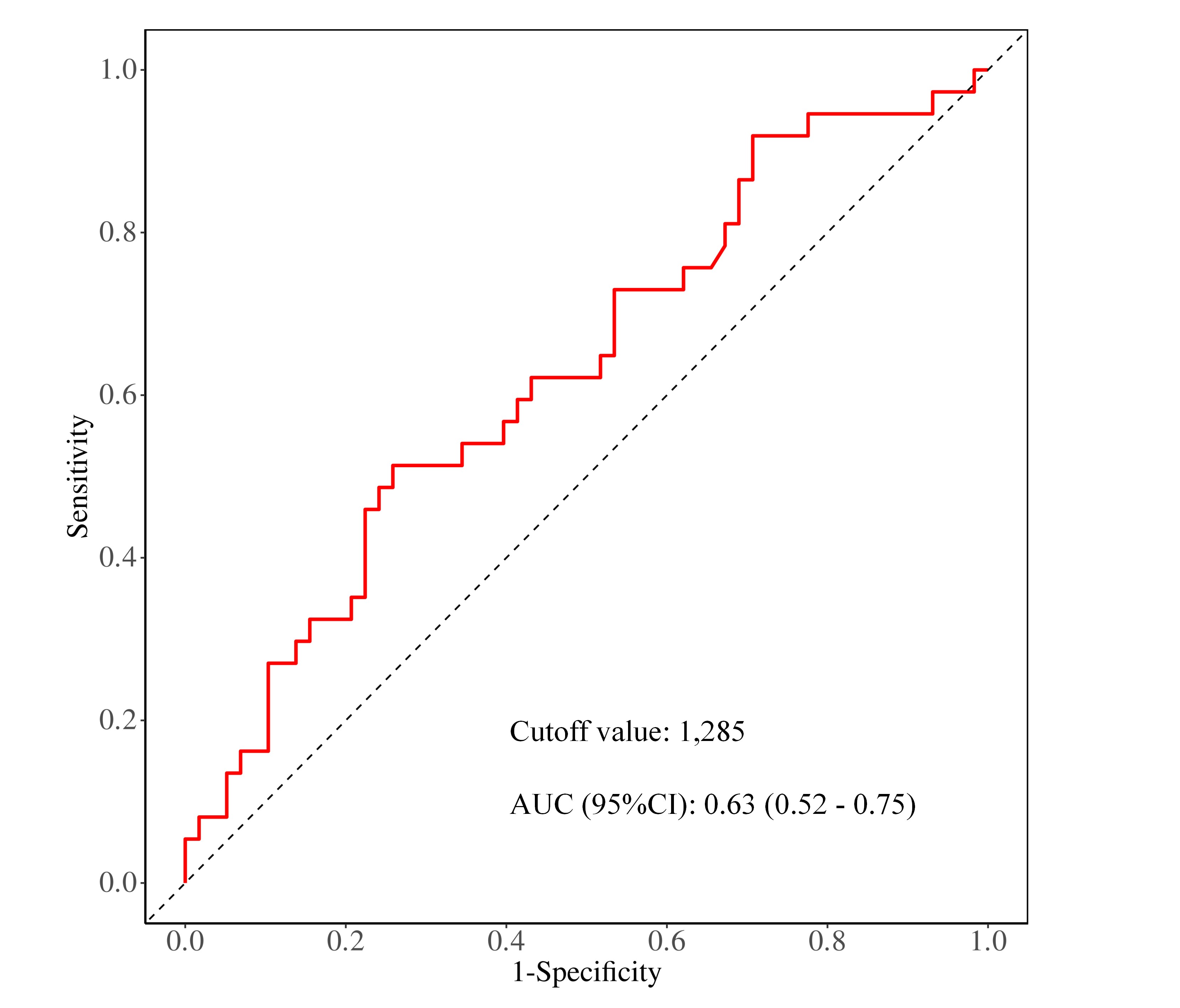

Supplement: Supplementary Figure 7 — ROC curve of D-dimer protein as a predictor of mortality in patients with first-episode iTTP. [file Image7.jpeg]

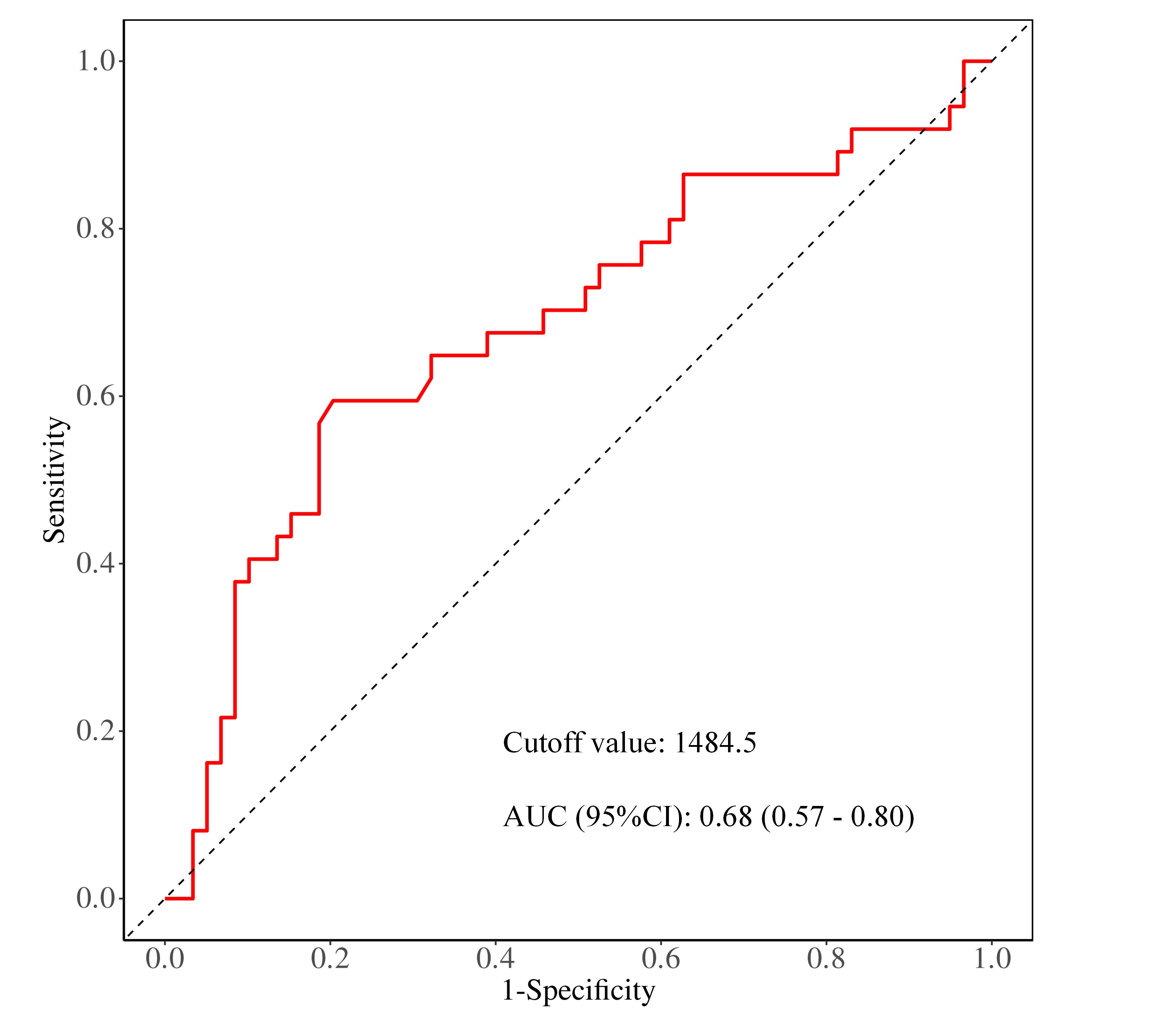

Supplement: Supplementary Figure 8 — ROC curve of lactate dehydrogenase as a predictor of mortality in patients with first-episode iTTP [file Image8.jpeg]

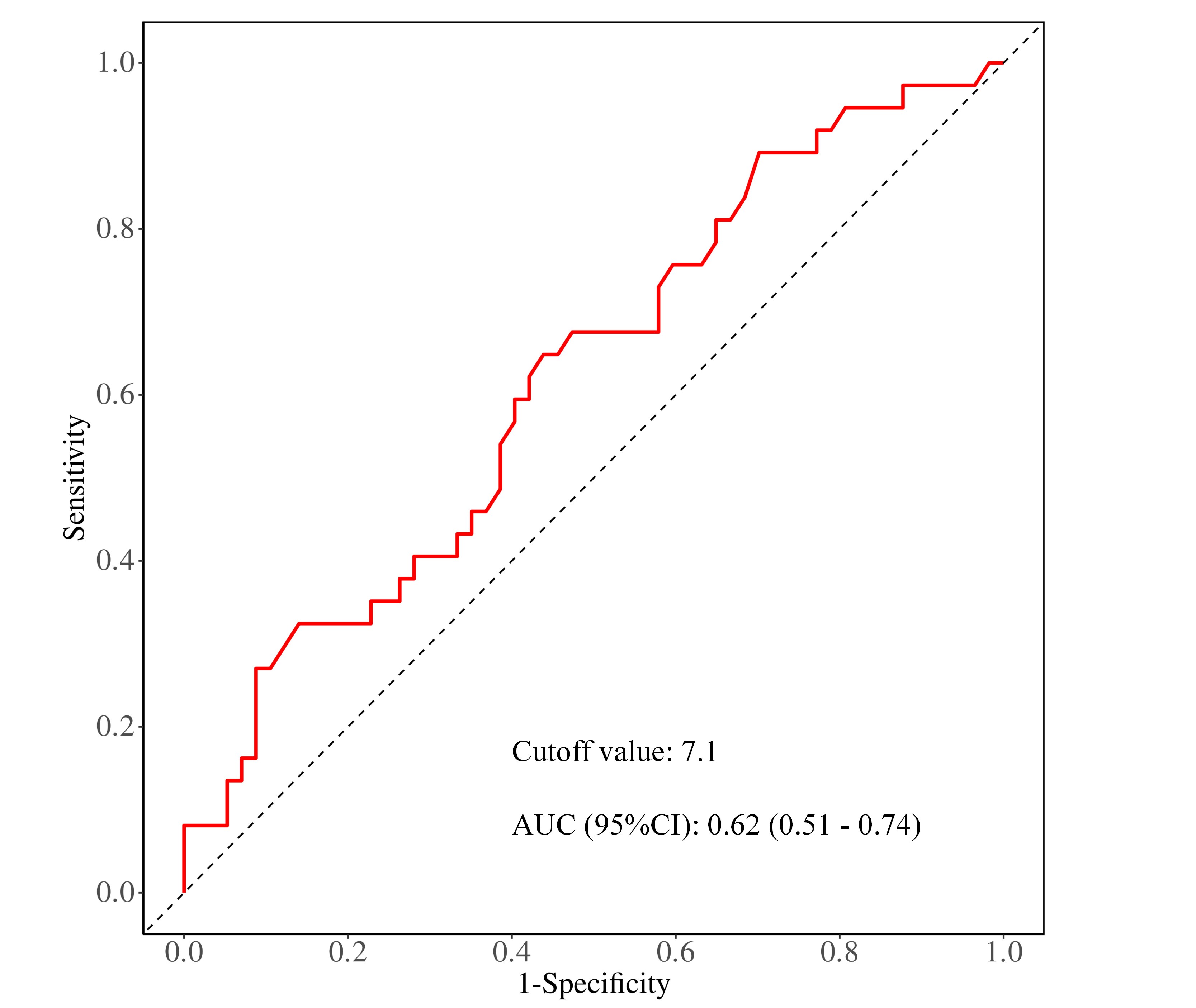

Supplement: Supplementary Figure 9 — ROC curve of fibrin degradation products as a predictor of mortality in patients with first-episode iTTP [file Image9.jpeg]
